# Dispersing hydrophobic natural colourant β-carotene in shellac particles for enhanced stability and tunable colour

**DOI:** 10.1098/rsos.170919

**Published:** 2017-12-13

**Authors:** Dong Chen, Chun-Xia Zhao, Camille Lagoin, Mingtan Hai, Laura R. Arriaga, Stephan Koehler, Alireza Abbaspourrad, David A. Weitz

**Affiliations:** 1 State Key Laboratory of Fluid Power and Mechatronic Systems, Zhejiang University, Zheda Road No. 38, Hangzhou, 310027, People's Republic of China; 2 Institute of Process Equipment, College of Energy Engineering, Zhejiang University, Zheda Road No. 38, Hangzhou, 310027, People's Republic of China; 3 John A. Paulson School of Engineering and Applied Sciences, Harvard University, Cambridge, MA 02138, USA; 4 Australian Institute for Bioengineering and Nanotechnology, The University of Queensland, St Lucia, Queensland 4702, Australia; 5 Department of Food Science, Cornell University, Ithaca, NY 14853, USA

**Keywords:** microfluidics, microencapsulation, colourant, β-carotene, microparticle

## Abstract

Colour is one of the most important visual attributes of food and is directly related to the perception of food quality. The interest in natural colourants, especially β-carotene that not only imparts colour but also has well-documented health benefits, has triggered the research and development of different protocols designed to entrap these hydrophobic natural molecules to improve their stability against oxidation. Here, we report a versatile microfluidic approach that uses single emulsion droplets as templates to prepare microparticles loaded with natural colourants. The solution of β-carotene and shellac in the solvent is emulsified by microfluidics into droplets. Upon solvent diffusion, β-carotene and shellac co-precipitates, forming solid microparticles of β-carotene dispersed in the shellac polymer matrix. We substantially improve the stability of β-carotene that is protected from oxidation by the polymer matrix and achieve different colour appearances by loading particles with different β-carotene concentrations. These particles demonstrate great promise for practical use in natural food colouring.

## 1. Introduction

Colour is used by consumers as an indicator of both the quality and safety of foods and people expect processed foods to be coloured attractively with shades that are typical of their product variety. Synthetic dyes are added to foods to improve their appearance, making them more appealing. However, these dyes may results in health issues such as sensitivity, intolerance and carcinogenicity [1]. They are thus preferentially avoided and substituted by natural colourants, such as β-carotene (E160a, the food additive code), which is the most widespread naturally sourced food colourant with health-promoting effects, the provitamin A activity and the antioxidant action [2,3]. β-Carotene has a strong red-orange colour due to its large sequence of conjugated double bonds [4]; however, the nature of double bonds makes β-carotene very sensitive to degradation, particularly to oxidation, which strongly limits its application. The oxidation of β-carotene includes firstly isomerization, secondly production of radical species, and then apparition of cleavage products [5,6]. The breakdown products include a number of small molecules with extremely low odour thresholds [7]. The use of β-carotene as a colourant is thus severely limited in food applications where the flavour profile is particularly mild [8].

Encapsulation has been widely used to remedy such problems by which certain sensitive ingredients are entrapped in a polymer matrix and protected from ambient conditions such as light, temperature, oxygen and humidity [9–17]. In general, the polymers must possess three critical features: they should be food grade, able to provide a good protection for the natural colourants and able to tune the property of the final product such as the colour appearance. Numerous efforts have been devoted to find the proper polymer matrix for β-carotene microencapsulation [18–20], and to develop the desired microencapsulation techniques [21–25]. The polymer matrices studied so far are mainly carbon hydrates; however, due to their hydrophilic nature, these polymers are not compatible with hydrophobic β-carotene. As a result, the protection provided by these polymers is not optimal because β-carotene cannot be well dispersed in the polymer matrix.

**Figure 1.**
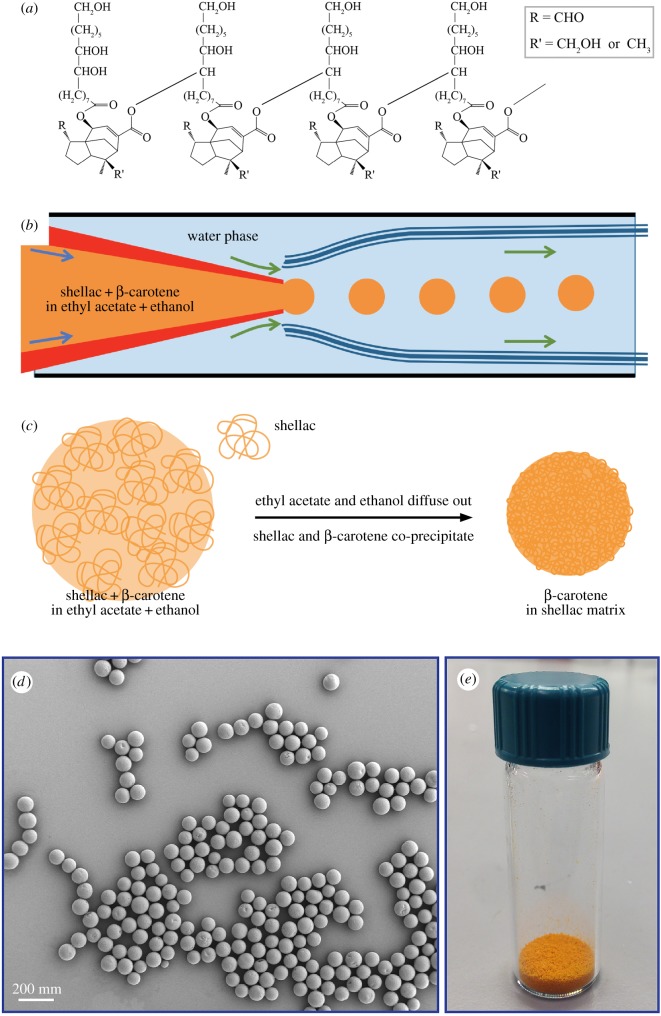
Production of monodisperse shellac particles loaded with β-carotene in the polymer matrix using single oil/water emulsions as templates. (*a*) The general chemical structures of shellac. (*b*) Schematic illustration of the glass capillary device used to produce monodisperse single emulsions in the dripping regime. (*c*) Schematic representation of solidification of shellac particles loaded with β-carotene upon solvent diffusion. (*d*) SEM image showing monodisperse shellac particles synthesized using single emulsions as templates. (*e*) Natural orange colour obtained by encapsulating β-carotene in the shellac matrix.

Here we use shellac (E904, the food additive code), a natural resin, to achieve the desired protection. Shellac is a hydrophobic polymer that consists of a complex mixture of polyhydroxy polycarboxylic esters, lactones and anhydrides [26,27], as shown in figure 1*a*, and is compatible with β-carotene. To encapsulate β-carotene, we develop microfluidic and solvent-diffusion techniques to fabricate shellac particles with β-carotene uniformly dispersed in the polymer matrix. We improve the stability of β-carotene substantially and control the colour appearance of the particles by tuning the β-carotene concentrations in the polymer matrix.

## 2. Materials and methods

### 2.1 Materials

β-Carotene is used as the food colourant (Type I, synthetic, ≥93% (UV), powder; Sigma-Aldrich, USA). Shellac is employed as the encapsulant (wax free, tested according to Ph. Eur.; Sigma-Aldrich, USA). β-Carotene and shellac are dissolved in a mixture of ethyl acetate (anhydrous, 99.8%; Sigma-Aldrich, USA) and ethanol (200 proof, anhydrous KOPTEC USP, Multicompendial; Koptec, USA). Nile red (TCI, Japan) is used as a β-carotene equivalent to visualize the distribution of hydrophobic molecules inside the shellac matrix using fluorescence microscopy. Polyvinyl alcohol (PVA, MW: 13 000–23 000 g mol^−1^, 87–89% hydrolysed; Sigma-Aldrich, USA) is dissolved in distilled water (Milli-Q system; Millipore Corporation, USA) and used as the outer phase in the emulsification device.

### 2.2 Methods

#### 2.2.1 Sample preparation

β-Carotene slowly degrades during storage. Undegraded β-carotene is insoluble in ethanol, while degraded β-carotene is soluble in ethanol. Therefore, β-carotene is purified before every use using ethanol as a washing solution due to the low solubility of β-carotene in it (0.03 mg ml^−1^). Washing steps are applied until the colour of the ethanol washing solution remains unchanged. Shellac is barely soluble in ethyl acetate. However, a small amount of ethanol in ethyl acetate, for example a 4 to 1 volume ratio, makes it a fair solvent for shellac and barely affects the solubility of β-carotene (0.5 mg ml^−1^). Therefore, 100 mg of shellac is dissolved in 1 ml of a mixture of ethyl acetate and ethanol at a 4 to 1 volume ratio, respectively. The shellac solution is then saturated with β-carotene. The solubility of β-carotene in the ethyl acetate/ethanol mixture is approximately of 0.5 mg ml^−1^. The β-carotene that remains undissolved is removed from the shellac solution using a filter with a typical pore size of 0.25 µm.

#### 2.2.2 Encapsulation

The experiments are carried out using glass microfluidic devices. Cylindrical capillaries (World Precision Instruments, Inc., USA) with inner and outer diameters of 0.58 and 1.0 mm, respectively, are used to fabricate the devices. These cylindrical capillaries are tapered to the desired size using a puller (Flaming/Brown Micropipette Puller, Model P-97; Sutter Instrument Co., USA). The tapered cylindrical capillaries are inserted into a square capillary (Atlantic International Technology, Inc., USA); the latter has an inner dimension only slightly larger than the outer diameter of the cylindrical capillaries, which facilitates their alignment. We use the glass capillary device to produce an emulsion: the inner phase consists of 0.5 mg ml^−1^ β-carotene and 100 mg ml^−1^ shellac in the ethyl acetate/ethanol mixture and it is pumped through the tapered cylindrical capillary; the outer phase consists of a 10 wt% polyvinyl alcohol (PVA) aqueous solution and it is pumped through the square capillary. All fluids are pumped into the microfluidic device using syringe pumps (Harvard PHD 2000 series; Harvard Apparatus, USA). Oil/water single emulsions are formed when the outer aqueous phase converges at the collection capillary. The resulting emulsions are collected in a water reservoir to rapidly solidify the particles as the solvent diffuses into the water phase.

#### 2.2.3 Morphology of the microparticles

The production of single emulsion drops is monitored using an inverted microscope (DM-IRB; Leica, USA) equipped with a fast camera (Phantom 9; Vision Research, USA). After microparticle preparation, optical images are obtained using an inverted microscope (TE2000-E; Melville, USA). Scanning electron microscope (SEM) images are obtained using an Ultra55 Field Emission Scanning Electron Microscope (FESEM Ultra55; Carl Zeiss, USA). The particle size is measured using ImageJ program and averaged over at least 60 microparticles. Confocal images are obtained using an inverted fluorescence microscope with an excitation wavelength of 485 nm (Leica, USA).

#### 2.2.4 Stability of the encapsulated material

After sample preparation, equal amounts of shellac particles loaded with β-carotene are stored in separate vials at 4°C. To measure the UV-vis absorption of β-carotene, 1 ml ethyl acetate/ethanol mixture is added to each vial. The concentration of undegraded β-carotene in each vial is determined by the UV-vis absorption of β-carotene in the ethyl acetate/ethanol mixture measured at 454 nm. The stability of microencapsulated β-carotene together with unprotected β-carotene is monitored at 0, 7, 35, 79 and 129 days. All experiments are performed in duplicate.

## 3. Results and discussion

### 3.1 Preparation of monodisperse colour particles

To prepare colour particles, we dissolve β-carotene and shellac together in an ethyl acetate/ethanol mixture, which is then emulsified into single drops using a flow-focusing glass capillary device, as shown in figure 1*b*. Droplet formation in the microfluidic device results from the Rayleigh instability [28]. In the dripping regime, monodisperse oil/water single emulsions are generated, as shown in electronic supplementary material, figure S1. Following droplet generation, ethyl acetate and ethanol continuously diffuse into the outer aqueous medium, resulting in the co-precipitation of shellac and β-carotene (figure 1*c*). The resultant solid, spherical microparticles consist of a shellac matrix uniformly loaded with β-carotene, as shown in figure 1*d*, and exhibit the desired orange colour, as shown in figure 1*e*.

**Figure 2.**
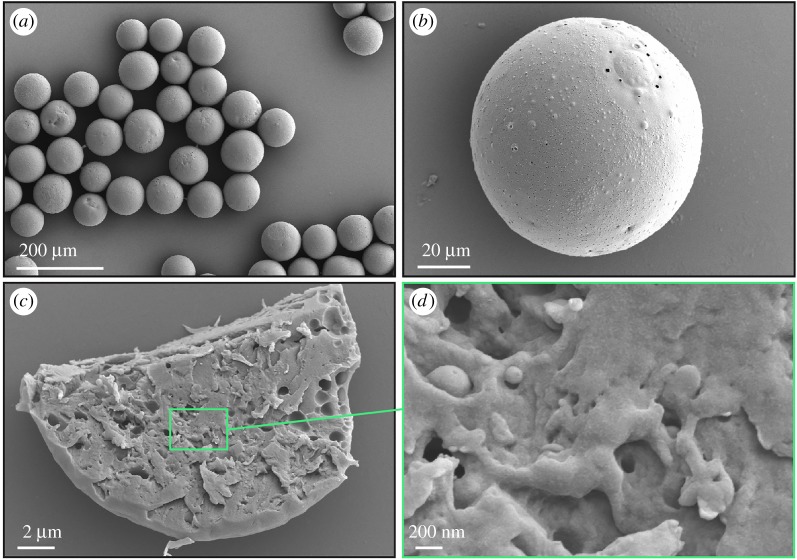
SEM images of monodisperse shellac particles loaded with β-carotene. (*a*,*b*) Monodisperse shellac particles achieved from single oil/water emulsions. (*c*) Cross section and (*d*) magnified image of a shellac particle. The polymer matrix formed by solvent diffusion is intense.

The advantage of using microfluidics is to control the drop size; this is particularly important in food applications, as the tongue is unable to resolve the texture of the particles provided they are smaller than 20 µm [29]. Since the particles are synthesized using single emulsions as templates, particles of desired size are achieved by controlling the size of single emulsions through adjusting the flow rate and changing the size of the device or varying the concentration of polymer in the single emulsions. We obtain monodisperse shellac particles with an average diameter of *d* ∼ 84 ± 6 µm using single emulsions of average size *d* ∼ 167 µm as templates, as shown in figure 2*a*. When we increase the flow rate of the outer aqueous phase to apply a stronger shear force to the inner phase and thus break it into smaller droplets, we obtain small shellac particles with an average size of *d* ∼ 19 ± 4 µm, which is satisfactory in the case where the texture of food needs to be smooth (electronic supplementary material, figure S2*a*–*d*).

### 3.2 Enhanced stability of encapsulated β-carotene

**Figure 3.**
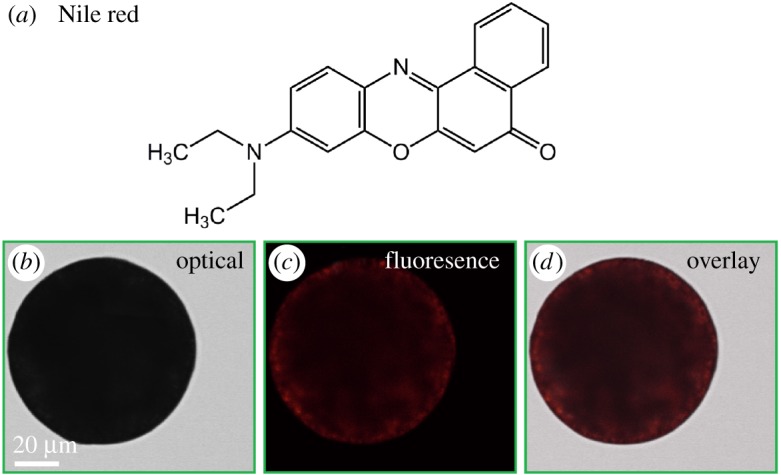
Distribution of hydrophobic molecules microencapsulated in the shellac matrix prepared by solvent diffusion. (*a*) Chemical structure of Nile red, a hydrophobic molecule used as an equivalent of β-carotene. (*b*) Optical, (*c*) fluorescence and (*d*) overlay images of shellac particles loaded with Nile red, which are prepared following the same procedure as used for β-carotene. The fluorescence colour suggests the uniform distribution of the hydrophobic molecules in the polymer matrix. The intensity of the red colour decreases towards the particle centre as less light transmits through the particle centre.

To achieve the desired protection, solid matrix is desired to exclude oxygen. We use microscopy to verify that our technique produces solid particles with β-carotene uniformly dispersed in a densely packed shellac polymer matrix, which is important to prevent oxidation of β-carotene. We cut the particles and image their cross section using SEM. The particles are solid with distinct cutting edges, as shown in figure 2*b*,*c*, and the polymer matrix is very dense, as shown by the close-up in figure 2*d*. To directly visualize the distribution of the colourants inside the polymer matrix, we use Nile red (figure 3*a*) as a β-carotene equivalent. Both Nile red and β-carotene are hydrophobic, insoluble in water and both of them are small molecules compared with the polymer. Therefore, we expect that the distribution of Nile red in the shellac particles is similar to that of β-carotene. We prepare shellac particles loaded with Nile red, following the same procedure as for β-carotene. The red colour of Nile red observed under fluorescent confocal microscope is homogeneously distributed inside the shellac matrix, as shown in figure 3*b–d*, suggesting that hydrophobic molecules are uniformly dispersed in the polymer matrix. We conclude that β-carotene is uniformly dispersed in the shellac particles and the dense polymer matrix is therefore expected to provide an effective barrier to β-carotene against the oxidation.

**Figure 4.**
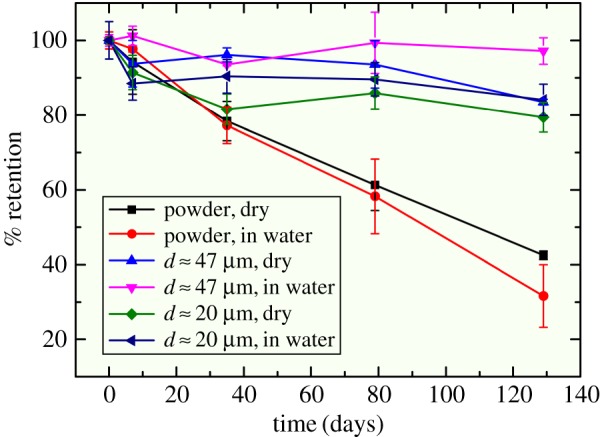
Retention of β-carotene microencapsulated in the shellac matrix over a long period of time. Retention of β-carotene protected by the shellac matrix is significantly higher than that in the control experiments. Dry shellac particles and shellac particles dispersed in water show roughly the same performance and the enhanced performance also has very little dependence on the particle size. For the purpose of comparison, the results are expressed in percentage.

To demonstrate the stability of β-carotene dispersed in the shellac particles, we monitor over time the fraction of undegraded β-carotene using UV-vis spectroscopy. When exposed to ambient conditions in the absence of any shellac protection, β-carotene degrades significantly within four months, as shown by the large decrease of the undegraded fraction in figure 4. In contrast, β-carotene dispersed in the shellac matrix is stable for a long period of time; we observe at least 80% of the encapsulated β-carotene remains intact and is not oxidized after four months also shown in figure 4. For these samples, most of the degradation occurs within the first week which is followed by a much slower degradation. This observation suggests that β-carotene close to the surface is more susceptible to oxidation than β-carotene deeper within the polymer matrix [30–32]. Since β-carotene is uniformly dispersed in the polymer matrix and protected from the ambient conditions, there is no obvious difference observed between the stability of β-carotene encapsulated in small (*d* ∼ 20 µm) and large (*d* ∼ 47 µm) particles. In addition, its stability is also independent of whether the particles are dry or wet. However, the stability of β-carotene generally decreases as the temperature increases [30]. Compared with previous techniques used to prevent the oxidation of β-carotene [33,34], the performance of the method introduced here is very good. For example, about 33% β-carotene encapsulated within nanoemulsions stabilized by β-lactoglobulin degraded within 6 days when stored at 5°C [33]. Therefore, the observed considerable reduction in degradation confirms the effectiveness of protecting β-carotene from oxidation by embedding within a dense polymer matrix.

Shellac is not soluble in acidic or neutral water, in which its carboxylic groups are only partially ionized, and these particles are stable [35]. However, shellac particles dissolve in alkaline water and β-carotene could thus be released in the intestine. We have observed that shellac particles dissolve at pH = 8 [35]. Therefore, shellac particles are able to protect β-carotene from degradation during storage and release β-carotene in the intestine when they are taken.

### 3.3 Particles with tunable colour

**Figure 5.**
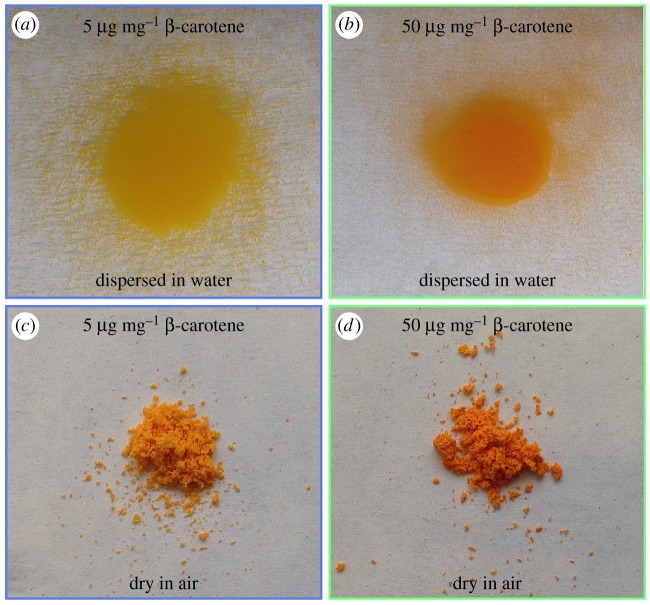
Different colour appearances of shellac particles loaded with different concentrations of β-carotene. Colour appearances of the particles loaded with (*a*) 5 µg mg^−1^ β-carotene (5 µg β-carotene per mg shellac) and (*b*) 50 µg mg^−1^ β-carotene when dispersed in water. (*c*) 5 µg mg^−1^ β-carotene and (*d*) 50 µg mg^−1^ β-carotene samples when dried in air.

The colours of the particles can be tuned by changing the concentration of β-carotene dispersed into the shellac matrix. Due to the long sequence of conjugated double bonds, β-carotene is a natural pigment that strongly absorbs blue and purple light, as shown in electronic supplementary material, figure S3. The absorbance is linearly proportional to the β-carotene concentration in solution and therefore obeys Beer--Lambert's Law, as shown in electronic supplementary material, figure S4. The colour that β-carotene displays arises from the intensities of transmitted wavelengths, while the absorbance by β-carotene is proportional to the concentration of β-carotene, as shown in the inset of electronic supplementary material, figure S3. Because β-carotene is uniformly dispersed in the shellac matrix, we expect that similar to solutions, the colour of particles is tunable by changing the concentration of β-carotene in the shellac matrix. For example, when we increase the concentration of β-carotene in the polymer matrix from 5 µg mg^−1^ (5 µg β-carotene per mg shellac) to 50 µg mg^−1^, the colour of the resultant particles changes from yellow orange (figure 5*a*) to red orange (figure 5*b*). The measured colour spectrum by Photoshop shows that the spectrum changes from yellow orange (Red:160, Green:110 and Blue:5) to red orange (Red:170, Green:80 and Blue:10) is mainly attributed to the increased adsorption of green colour by β-carotene at higher concentration. The difference in colour is also apparent in dried samples, as shown in figure 5*c*,*d*. We have demonstrated that the colour appearance of these particles can be tailored by selectively changing the β-carotene concentration, which makes them suitable for a range of natural colourants.

## 4. Conclusion

A current challenge for the food industry is replacing synthetic colour with natural alternatives, because natural colourants are generally sensitive to light, temperature, pH and redox agents. We demonstrate that shellac, which is of hydrophobic nature, is compatible with the hydrophobic β-carotene; this allows us to uniformly disperse β-carotene in the polymer matrix that constitutes the microparticles. The designed shellac particles prevent β-carotene from degradation during storage and show tunable colour when loaded with different β-carotene concentrations. We speculate that encapsulating with a sacrificial antioxidant such as caffeine or propyl gallate will further improved β-carotene's stability. The ability to extend the microfluidic technique to spray drying makes these colour particles feasible for industrial mass production and further facilitates the industrial application of these particles (electronic supplementary material, figures S5*a*–*i* and S6*a*–*i*). The capability of shellac particles to implement a diverse set of natural colourants that are generally hydrophobic should make them valuable for natural food colouring. Our work thus represents an important step towards the fabrication of microencapsulated natural food colourants with extended shelf life and tunable colour.

## Supplementary Material

Supporting Information

## Data accessibility

The datasets supporting this article have been uploaded as part of the electronic supplementary material.
