# Supplementary material for: Dispersing hydrophobic natural colourant β-carotene in shellac particles for enhanced stability and tunable colour
Source: R Soc Open Sci. 2017 Dec 13;4(12):170919. doi: 10.1098/rsos.170919 (PMC5750000; doi:10.1098/rsos.170919)
Supplement: Supporting Information [file rsos170919supp1.doc]

**Supplementary Information for
Dispersing Hydrophobic Natural Colorant β-carotene in Shellac Particles for Enhanced Stability and Tunable Color**

Dong Chen^a,b,c^, Chun-Xia Zhao^c,d^, Camille Lagoin^c^, Mingtan Hai^c^, Laura Arriaga^c^, Stephan Koehler^c^, Alireza Abbaspourrad^c,e*^ and David A Weitz^c*^

^a^State Key Laboratory of Fluid Power and Mechatronic Systems, Zhejiang University, Zheda Road No. 38, Hangzhou, 310027, China

^b^Institute of Process Equipment, College of Chemical and Biological Engineering,
Zhejiang University, Zheda Road No.38, Hangzhou, 310027, China

^c^John A. Paulson School of Engineering and Applied Sciences, Harvard University,
Cambridge, Massachusetts, 02138, USA

^d^Australian Institute for Bioengineering and Nanotechnology, The University of Queensland, St Lucia, QLD, Australia 4072

^e^Department of Food Science, Cornell University, Ithaca, NY, 14853, USA

^＊^E-mail: [alireza@cornell.edu](mailto:alireza@cornell.edu); [weitz@seas.harvard.edu](mailto:weitz@seas.harvard.edu)


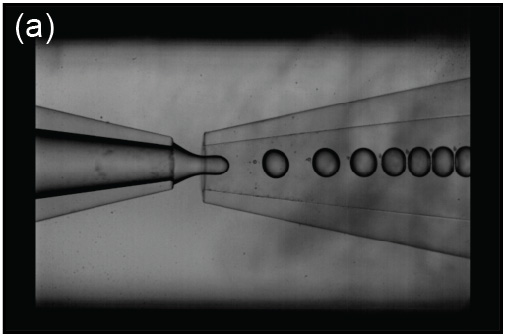


Figure S1: Real time image of the glass capillary device generating monodisperse single emulsions in the dripping region with an inner phase flow rate of 80 μl/hr and an outer phase flow rate of 500 μl/hr.


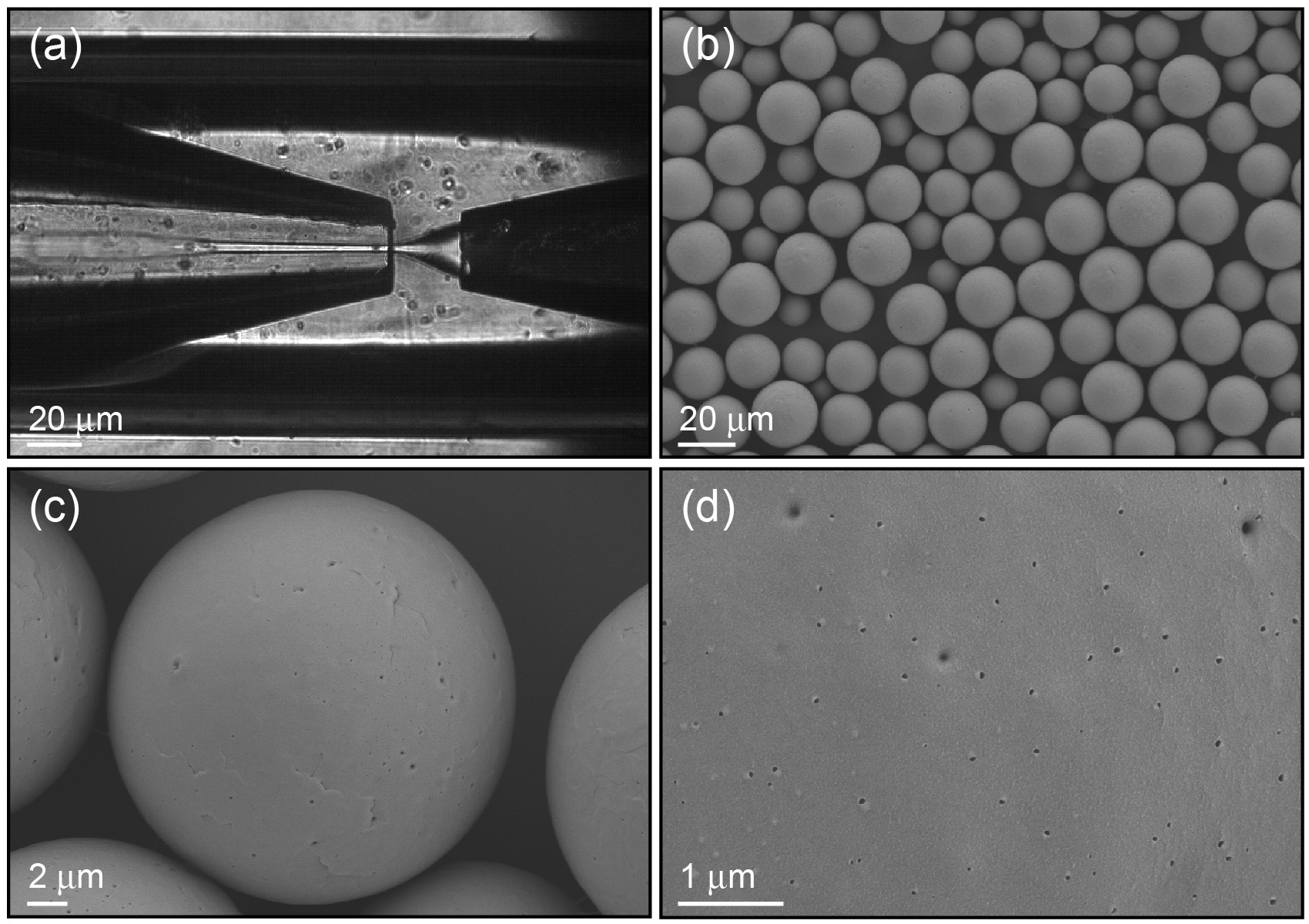


Figure S2: SEM images of small shellac particles. (a) In situ generation of small droplets in the jetting region with an inner phase flow rate of 80 μl/hr and an outer phase flow rate of 15000 μl/hr. (b), (c) and (d) SEM images of solidified particles taken at different magnification.


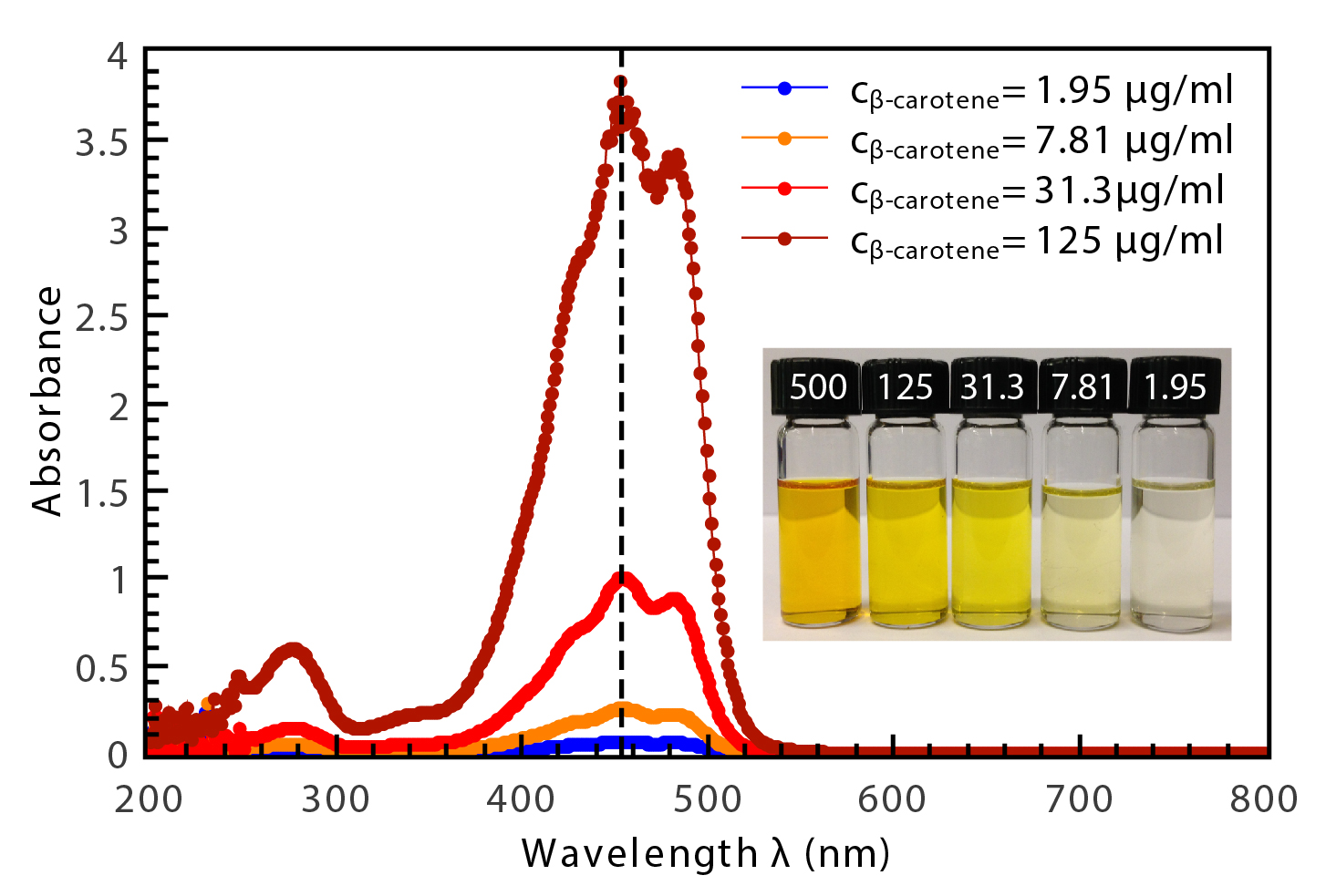


Figure S3: Absorptions of β-carotene in the ethyl acetate/ethanol mixture. The absorption strength depends on the concentration of beta-carotene in the ethyl acetate/ethanol mixture. The inset shows their corresponding color appearances.


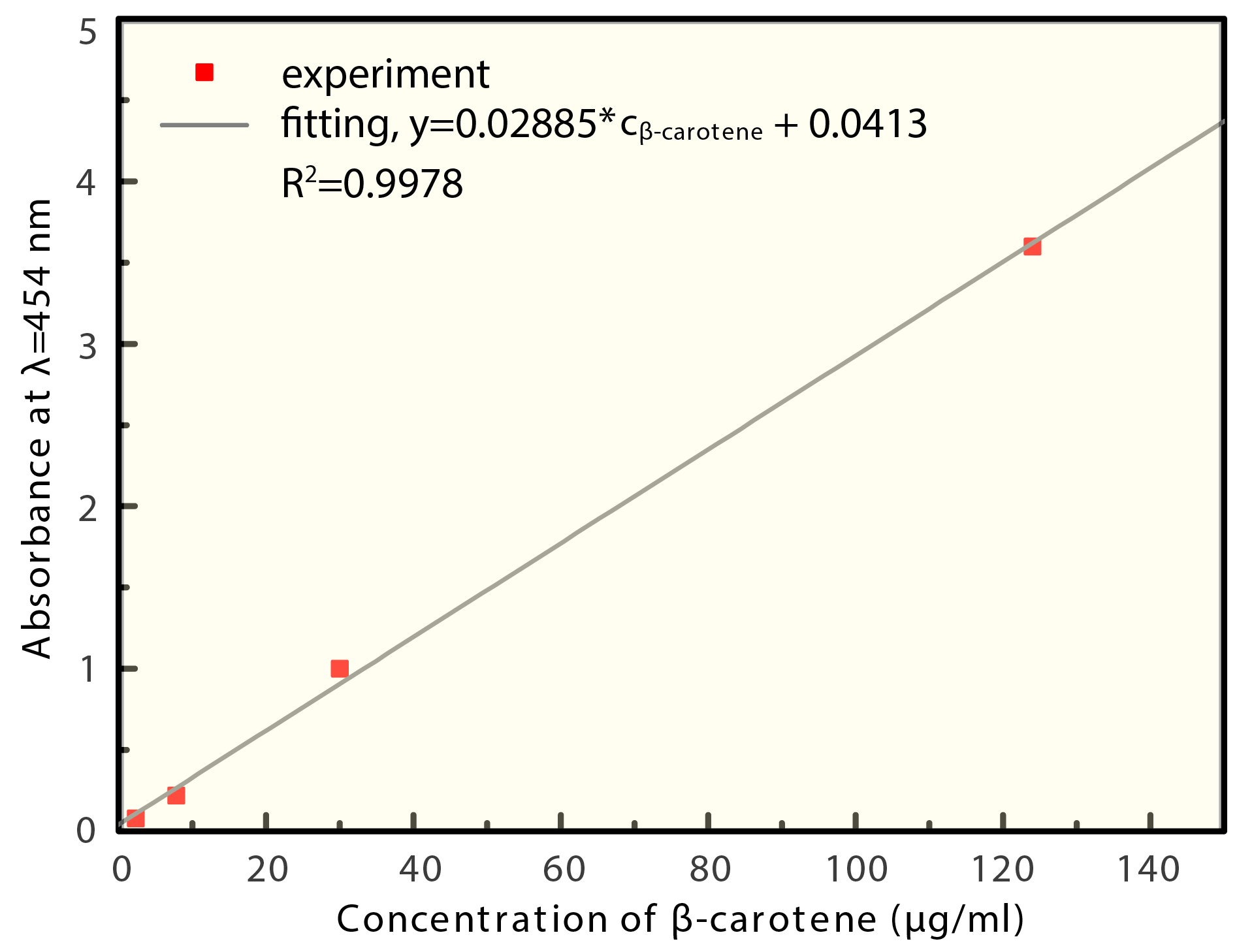


Figure S4: Linear dependence of the absorption strength on the β-carotene concentration. The absorption is measured at 454 nm, which follows the Beer-Lambert law.


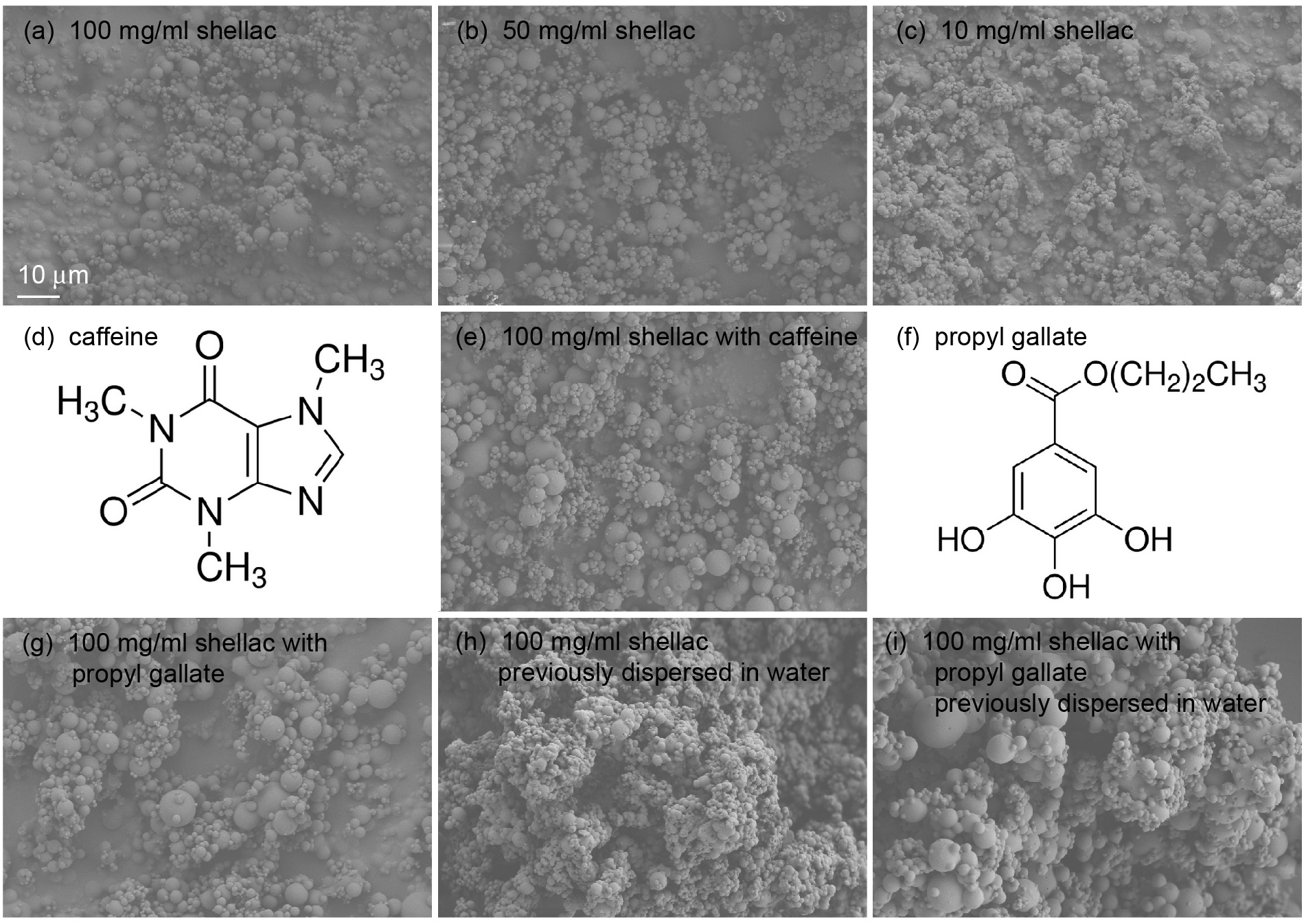


Figure S5: Solutions of β-carotene and shellac in mixtures of ethyl acetate and ethanol are fed into a spray dryer and atomized. The solvents are evaporated by the hot air contracting the atomized drops. SEM images (3000 times magnification) of spray-dried samples prepared from solutions of different shellac concentrations. (a) 100 mg/ml shellac. (b) 50 mg/ml shellac. (c) 10 mg/ml shellac. (d) Molecular structure of caffeine. (e) 100 mg/ml shellac with 1 mg/ml caffeine. (f) Molecular structure of propyl gallate. (g) 100 mg/ml shellac with 1 mg/ml propyl gallate. (h) and (i) Shellac particles that are previously dispersed in water. There is no substantial size change when the concentration of shellac decreases from 100 mg/ml to 50 mg/ml; however, the particles are obviously smaller when it decreases to 10 mg/ml.


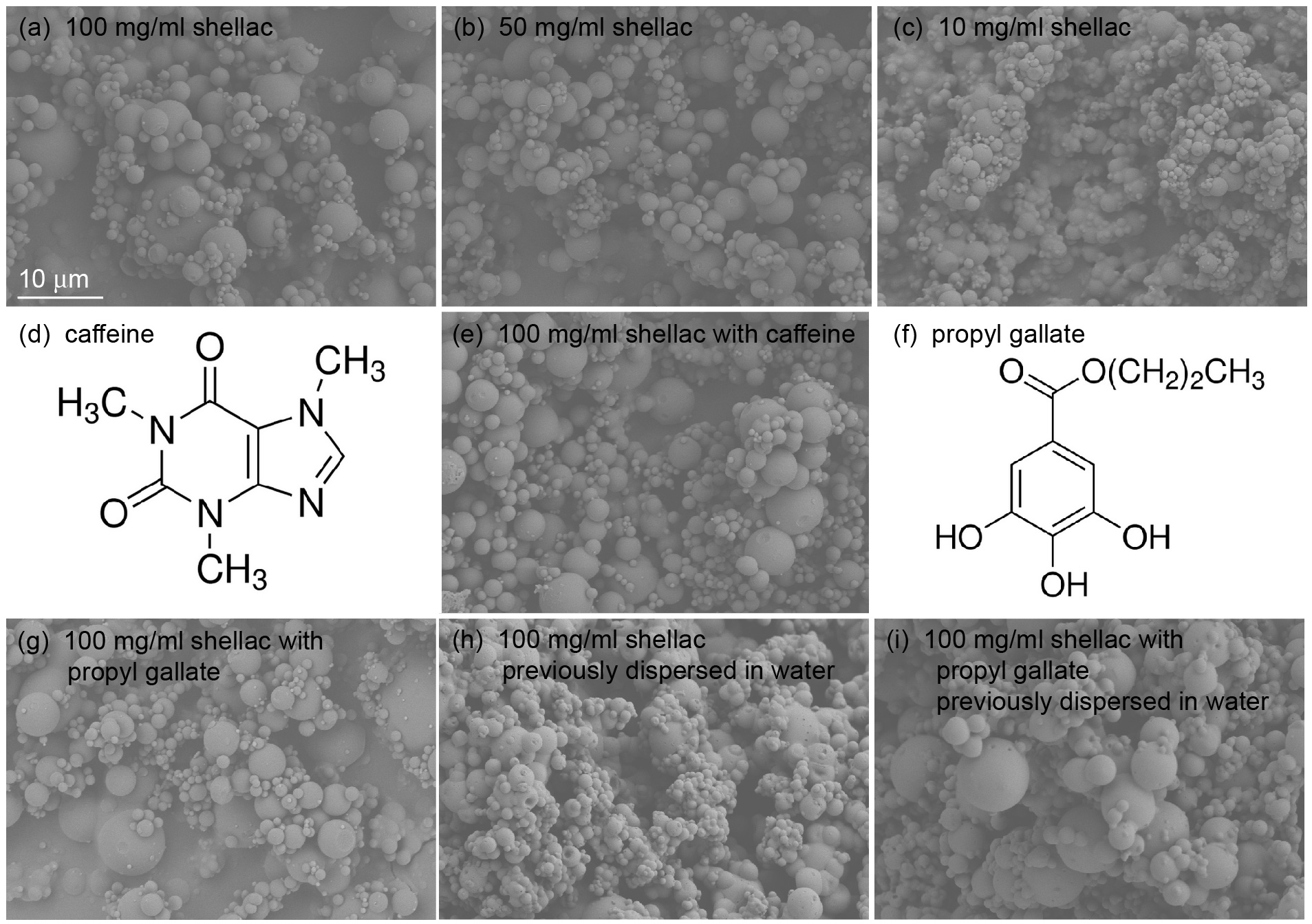


Figure S6: SEM images (6000 times magnification) of spray-dried samples prepared from solutions of different shellac concentrations. (a) 100 mg/ml shellac. (b) 50 mg/ml shellac. (c) 10 mg/ml shellac. (d) Molecular structure of caffeine. (e) 100 mg/ml shellac with 1 mg/ml caffeine. (f) Molecular structure of propyl gallate. (g) 100 mg/ml shellac with 1 mg/ml propyl gallate. (h) and (i) Shellac particles that are previously dispersed in water.
